# Antineutrophil Cytoplasmic Antibody-Associated Vasculitis Update: Genetic Pathogenesis

**DOI:** 10.3389/fimmu.2021.624848

**Published:** 2021-03-26

**Authors:** Weiran Li, He Huang, Minglong Cai, Tao Yuan, Yujun Sheng

**Affiliations:** ^1^ Institute of Dermatology and Department of Dermatology, The First Affiliated Hospital, Anhui Medical University, Hefei, China; ^2^ Key Laboratory of Dermatology, Anhui Medical University, Ministry of Education, Hefei, China

**Keywords:** vasculitis, antineutrophil cytoplasmic antibody, genetic, genome-wide association studies, variation

## Abstract

Antineutrophil cytoplasmic antibody (ANCA)-associated vasculitis (AAV) is characterized by the inflammation of small and medium vessels and presence of proteinase 3-ANCA or myeloperoxidase-ANCA in the circulation. AAV comprises three clinical subtypes: granulomatosis with polyangiitis (GPA), microscopic polyangiitis (MPA), and eosinophilic GPA (EGPA). Although the pathogenesis of AAV is still unclear, genetic and environmental factors and the immune system are thought to be involved. Genetic factors have been confirmed to play an important role in AAV. Genome-wide association studies have identified numerous genetic variants in MHC and non-MHC regions associated with AAV. The strongest evidence of MHC association in AAV is human leukocyte antigen (HLA)-DP. A significant association between AAV and genetic variations in non-MHC regions, such as *CTLA-4*, *FCGR2A*, *PTPN22*, *SERPINA1*, and *TLR9* has also been found. Moreover, different clinical subtypes of AAV have distinct genetic backgrounds. GPA is associated with *HLA-DP1*, MPA with *HLA-DQ*, and EGPA with *HLA-DRB4*. These findings could help elucidate the etiology of AAV and develop new biomarkers for diagnosis and targeted therapy. Herein, we briefly summarize the updates on the genetic pathogenesis and biomarkers of AAV.

## Introduction

Antineutrophil cytoplasmic antibody (ANCA)-associated vasculitis (AAV) is a complex systemic autoimmune disease presenting with the inflammation of small and medium vessels that results in vascular destruction and tissue necrosis (1, 2). AAV is divided into three groups according to clinical features: granulomatosis with polyangiitis (GPA, formerly called Wegener’s granulomatosis), microscopic polyangiitis (MPA), and eosinophilic GPA (EGPA, formerly called Churg-Strauss syndrome) (3). The disease is characterized by the presence of proteinase 3 (PR3)-ANCA or myeloperoxidase (MPO)-ANCA in the serum. GPA is predominantly associated with PR3-ANCA, while MPA and EGPA are predominantly associated with MPO-ANCA, but are also occasionally ANCA-negative (4). EGPA is often divided into two subtypes, MPO-ANCA+ EGPA and ANCA– EGPA. Vasculitis can occur in any organ or tissue, commonly affecting the respiratory tract and kidneys, causing life-threatening kidney failure or pulmonary hemorrhage (3).

AAV has an estimated prevalence of 48–184/1,000,000 individuals worldwide, while the incidence and prevalence in Europe are higher than those in other regions (2). The annual incidence in Europe is 4.9–10.6/1,000,000 individuals for GPA, 2.7–11.6/1,000,000 individuals for MPA, and 0.5–3.1/1,000,000 individuals for EGPA. The epidemiological manifestations of AAV differ among geographical regions; GPA and PR3-ANCA AAV are more common in Europeans, while MPA and MPO-ANCA AAV are more common in Asians (5).

Although the pathogenesis of AAV remains elusive, it is believed that both genetic and environmental components are involved. Environmental factors, including silica exposure, bacterial or viral infections, and drugs suggest an association with the occurrence and relapse of AAV (6). Familial studies and genetic association studies have demonstrated that AAV has a background of genetic susceptibility (7–9). It has been estimated that 20% of AAV risk is due to genetic factors (10).

In this review, we mainly discuss the genetic studies on AAV, focusing on the identified susceptibility genes or loci to enrich our understanding of the disease.

## Genetic Approaches for AAV

Different genetic approaches have revealed that a large number of genes are related to AAV. First, several candidate gene association studies have been used to identify susceptibility genes associated with AAV. Although these approaches are easy to carry out, they are prone to false-positive results. Human leukocyte antigen *(HLA)*, *PTPN22*, *CTLA*-*4*, *IL-10*, and *TLR9* have been found to be associated with AAV (11, 12). The emergence of genome-wide association studies (GWAS) has led to their wide use in exploring the genetic factors involved in various diseases. GWAS is a powerful approach to identify the genetic architecture of complex diseases, and it has made major contributions towards a better understanding of AAV genetics. Four GWAS have been performed in patients with AAV since 2012 (two for MPO and GPA (13, 14), one for GPA (15), and one for EGPA (16)). The latest GWAS was on EGPA in 676 cases and 6809 controls with the identification of 11 loci associated with EGPA. The study also revealed that EGPA comprises genetically distinct subgroups, that MPO-ANCA+ subgroup was strongly associated with HLA-DQ and MPO-ANCA- subgroup was associated with non-HLA regions, such as GPA33 and IL5/IRF1 (16). All these GWAS studies were conducted in populations with European ancestry and have identified more than 20 genes associated with AAV. The findings of GWAS are listed in **Tables 1** and **2**. In addition, meta-analysis and fine mapping have also been used to identify new gene variations and analyze the effect of the variants (17–21).

**Table 1:** A summary of main genetic associations with antineutrophil cytoplasmic antibody (ANCA)-associated vasculitis through genome-wide association studies, according to Clinical subgroups.

Subgroups	Chr	Reported Gene(s)	SNP	Population	Cases-Controls	P-value	OR	Reference (PMID)
GPA and MPA	1p13.2	*PTPN22*	rs2476601	European	1986-4723	1.86×10^−7^	1.36	28029757
	1p13.2	*PTPN22*	rs6679677	European	1986-4723	1.88×10^−8^	1.4	28029757
	6p21.32	*HLA-DP*	rs3117242	European	2267-6858	1.5×10^−71^	3.67	22808956
	6p21.32	*COL11A2*	rs3130233	European	2267-6858	7.8×10^−15^	1.51	22808956
	6p21.32	*COL11A2*	rs3117016	European	2267-6858	6.4×10^−24^	1.83	22808956
	6p21.32	*SERPINA1*	rs7151526	European	2267-6858	2.4×10^−9^	0.59	22808956
	19p13.3	*PRTN3*	rs62132295	European	1353-1599	7.1×10^−5^	0.78	22808956
	6p21.32	*HLA–DPB1*	rs141530233	European	1986-4723	1.13×10^−89^	2.99	28029757
	6p21.32	*HLA–DPB1*	rs1042169	European	1986-4723	1.12×10^−84^	2.82	28029757
	6p21.32	*HLA–DPA1*	rs9277341	European	1986-4723	6.09×10^−71^	2.44	28029757
	6p21.32	*HLA–DQA1*	rs35242582	European	1986-4723	6.34×10^−23^	1.6	28029757
	6p21.32	*HLA–DQB1*	rs1049072	European	1986-4723	6.46×10^−13^	1.4	28029757
	6p21.32	*SERPINA1*	rs28929474	European	1986-4723	3.09×10^−12^	2.18	28029757
	19p13.3	*PRTN3*	rs62132293	European	1986-4723	8.60×10^−11^	1.29	28029757
GPA	5q23.1	*SEMA6A*	rs26595	European	987-2731	2.9×10^−8^	0.74	23740775
	6p21.3	*HLA–DPA1*	rs9277341	European	750-1820	2.18×10^−39^	0.33	23740775
	6p21.3	*HLA–DPB1*	rs9277554	European	750-1820	1.92×10^−50^	0.24	23740775
	6p21.32	*HLA-DP*	rs3117242 (G)	European	1683-6858	3.1×10^−85^	5.39	22808956
	6p21.32	*HLA–DPB1*	rs141530233	European	1556-4723	3.8×10^-93^	3.82	28029757
	6p21.32	*HLA–DPB1*	rs1042169	European	1556-4723	1.09×10^−90^	3.66	28029757
	6p21.32	*HLA–DPA1*	rs9277341	European	1556-4723	2.78×10^-73^	2.86	28029757
	6p21.32	*HLA–DQA1*	rs35242582	European	1556-4723	1.6×10^−20^	1.63	28029757
	6p21.32	*SERPINA1*	rs7151526	European	1683-6858	4.4×10^−10^	0.54	22808956
	6p21.32	*SERPINA1*	rs28929474	European	1556-4723	3.53×10^−13^	2.35	28029757
	19p13.3	*PRTN3*	rs62132293	European	1556-4723	7.06×10^−11^	1.32	28029757
	Xp22.2	*MOSPD2*	rs6628825	European	1683-6858	2.6×10^−5^	0.8	22808956
MPA	6p21.32	*HLA–DQB1*	rs1049072	European	236-4723	4.16×10^-9^	1.89	28029757
EGPA	2q13	*BCL2L11*	rs72946301	European	534-6809	9.0 × 10^−11^	1.81	31719529
	5q22.1	*TSLP*	rs1837253	European	534-6809	5.2 × 10^−11^	1.52	31719529
	6p21.32	*HLA-DQ*	rs9274704	European	534-6809	1.2 × 10^−20^	2.01	31719529
	10p14	*10p14*	rs34574566	European	534-6809	2.9 × 10^−8^	0.7	31719529

**Table 2:** Summary of main genetic associations with antineutrophil cytoplasmic antibody (ANCA)-associated vasculitis through genome-wide association studies, according to ANCA subgroups.

Subgroups	Chr	Reported Gene(s)	SNP	Population	Cases-Controls	P-value	OR	Reference
PR3+ AAV	6p21.32	*HLA−DP*	rs3117242	European	1521-6858	6.2×10^−89^	7.03	22808956
	6p21.32	*HLA–DPB1*	rs141530233	European	1361-4723	1.33×10^-106^	6.19	28029757
	6p21.32	*HLA–DPB1*	rs1042169	European	1361-4723	6.53×10^−106^	6.09	28029757
	6p21.32	*HLA–DPA1*	rs9277341	European	1361-4723	4.52×10^-84^	3.69	28029757
	6p21.32	*HLA–DQA1*	rs35242582	European	1361-4723	5.78×10^−18^	1.62	28029757
	6p21.32	*SERPINA1*	rs28929474	European	1361-4723	1.29×10^−13^	2.43	28029757
	19p13.3	*PRTN3*	rs62132295(A)	European	1521-1599	2.6×10^−7^	0.73	22808956
	19p13.3	*PRTN3*	rs62132293	European	1361-4723	3.59×10^−13^	1.39	28029757
	6q22.33	*ARHGAP18*	rs1705767	European	1521-6858	5.2×10^−8^	0.73	22808956
	14q32.13	*SERPINA1*	rs7151526	European	1521-6858	5.6×10^−12^	0.53	22808956
	Xp22.2	*MOSPD2*	rs6628825	European	1521-6858	6.1×10^−7^	0.77	22808956
MPO+ AAV	6p21.32	*HLA-DQ*	rs5000634	European	556-6858	2.1×10^−8^	0.65	22808956
	6p21.32	*HLA–DQA2*	rs3998159	European	378-4723	5.24×10^-25^	2.72	28029757
	6p21.32	*HLA–DQA2*	rs7454108	European	378-4723	5.03×10^-25^	2.73	28029757
MPO- AAV	6p21.32	*HLA–DQB1*	rs1049072	European	378-4723	2.13×10^-24^	2.37	28029757

## AAV susceptibility genes

### HLA Region

#### HLA

*HLA*, located on chromosome 6p21, is the most gene-dense region of the human genome and contains diverse genes involved in key immune responses (22). Molecules encoded by *HLA* participate in a variety of immune and inflammatory pathways. *HLA* single-nucleotide polymorphisms (SNPs) are associated with numerous human diseases, especially autoimmune diseases, such as type 1 diabetes, rheumatoid arthritis (RA), and systemic lupus erythematosus (SLE) (23). Since AAV is considered an autoimmune disease, *HLA* may also be a potential predisposing factor for AAV. Candidate gene studies in *HLA* regions were performed in different populations, including the Swedish, Germans, and Italians in Europe and Japanese and Chinese in Asia. *HLA-DPA1*, *HLA-DPB1*, *HLA-DQA1*, *HLA-DQB1*, and *HLA-DRB1* were found to be involved in AAV susceptibility.

(1). HLA and AAV
 Two GWAS were performed to identify the genetic factors associated with AAV. The first GWAS in a European population in 2012 demonstrated that *HLA-DP* rs3117242 (G) was the strongest signal in the HLA region (*P* = 1.5×10^−71^, OR = 3.67) (13). Another GWAS in Canadian and American populations conducted in 2017 showed that SNPs rs141530233 and rs1042169 at *HLA-DPB1* had the largest effect on the risk of developing AAV (*P* = 1.13×10^−89^, OR = 2.99; *P* = 1.12×10^-84^, OR = 2.82, respectively). *HLA-DPA1*, *HLA-DQA1*, and *HLA-DQB1* are also risk alleles that may cause AAV (14). These studies not only indicated a highly significant association between AAV and *HLA* regions but also showed genetic distinctions between different clinical phenotypes and ANCA specificity. GPA and PR3-ANCA AAV are associated with *HLA-DPB1* and *HLA-DPA1*, while MPA and MPO-ANCA AAV are associated with *HLA-DQB1* and *HLA-DQA2*.
(2). HLA and GPA
 GWAS for GPA with 492 patients and 1,506 healthy individuals of European descent identified 32 SNPs across the HLA region; among them, *HLA-DPB1* rs9277554 and *HLA-DPA1* rs9277341 were significantly associated with GPA in the combined cohort (15). In another study with 150 GPA patients and 100 healthy controls conducted in northern Germany, HLA-DPB1*0401 was identified to be associated with GPA (*P* = 1.51 × 10^-10^, OR = 3.91), and DPB1*0401/RXRB03 haplotype frequency was significantly increased in patients with GPA (*P* = 7.13 × 10^-17^, OR = 6.41) (18). The result was replicated in an independent German cohort with 108 patients with GPA (*P* = 6.4 × 10^-8^) (20). *HLA-DPB1* was also considered a genetic risk factor for GPA in a cohort of 176 Han Chinese patients with AAV (100 with GPA, 76 with MPA) and 485 healthy controls (*P* = 1.83 × 10^-5^, OR = 2.57). In this study, the result also showed that the *HLA-DPB1* SNP rs3117242 variant T allele was significantly associated with GPA patients (P = 6.24 × 10^-5^, OR = 2.09), but not with MPA patients (24).
(3). HLA and MPA
 There was no specific GWAS study for MPA, but the clinical subgroup analysis of AAV GWAS demonstrated that *HLA-DQB1* was significantly associated with MPA (*P* = 4.16×10^−9^, OR = 1.89) (14). A significant association of HLA-DRB1*0901 with MPA (*P* = 0.0037, OR = 2.44) and MPO-AAV (*P* = 0.0014, OR = 2.44) was demonstrated in 50 MPA and 64 MPO-ANCA AAV cases compared with 265 controls in a Japanese cohort (25). The result was confirmed with new observations that DRB1*13:02 was associated with protection against MPA and MPO-ANCA AAV in 468 Japanese patients with AAV and 596 controls(P = 2.1 × 10^-4^, OR = 1.57) (26). In a small cohort of 50 patients with MPA and 77 Japanese controls, DQB1*0303 was significantly associated with MPA (*P* = 0.017, OR = 2.35) (27). A recent study genotyped *HLA-DRB1*, *DQA1*, *DQB1*, *DPB1*, and *HLA-DP* in 258 patients with MPO-AAV and 597 healthy controls in the Chinese population and found that HLA-DQA1*0302 (*P* = 3.45 × 10^-9^, OR = 2.34) and DQB1*0303 (*P* = 3.26 × 10^-9^, OR = 1.89) were risk alleles for MPO-ANCA AAV (28).
(4). HLA and EGPA
 A recent GWAS for EGPA including 676 EGPA cases and 6,809 controls in European populations showed that *HLA-DQ* was strongly associated with EGPA at genome-wide significance (*P* = 1.2 × 10^−20,^OR = 2.01), especially MPO -ANCA EGPA (*P* = 1.1 × 10^−28^, OR = 5.68) (16). This result was consistent with a study comprising 102 German patients with EGPA (*P* = 2.00×10^−5^, OR = 1.87) (29) as well as another study with 48 Italian patients (*P* = 2.32 × 10^−4^, OR = 2.49) (30). The Italian study also revealed that HLA-DRB1*07 allele frequency was significantly higher in patients with EGPA than in controls (*P* = 0. 42 × 10^−2^, OR = 2.42) (30).
 Furthermore, *HLA* alleles were associated with the severity, prognosis, and relapse of AAV. HLA-DRB1*04:05 was associated with poor renal prognosis; HLA-DRB1*04:02 was associated with high mortality (31); and DPB1*04:01 was significantly associated with an increased risk of relapse (32).
 AAV is an autoimmune disease. The identification of HLA-alleles associated with AAV confirm the centrality of auto-reactivity in the development of AAV, help us make a better understanding about its autoimmune pathogenesis, and point to logical therapeutic strategies. The identification of HLA-alleles not only can help us to distinguish from cases of GPA, MPA and EGPA and their subtypes, but also to evaluate and predict the severity, prognosis, and relapse of AAV.

#### Retinoid X Receptor Beta

*RXRB*, located on 6p21.32, encodes a member of the retinoid X receptor family of nuclear receptors. Strong associations of *RXRB* with GPA were revealed through the markers located in the *HLA* region and *RXRB* loci. Because *RXRB* located in the *HLA* region near *HLA-DPB1*, HLA-DPB1*0401/RXRB03 haplotype was found to be strongly associated with GPA (*P* = 7.13 × 10^-17^, OR = 6.41) (18). *RXRB* SNP rs6531 (*P* = 5.20 × 10^-5^, OR = 1.88) was significantly increased among patients with GPA compared to controls (19). The result was confirmed in a later meta-analysis, which included 140 genetic variants associated with AAV (17).

#### Ring Finger Protein 1

*RING* is also located in the *HLA* region, near *HLA-DPB1*. RING is a member of the polycomb repressive complex 1 that mediates histone H2A polyubiquitination and monoubiquitination, regulating its gene expression. Three SNPs in the region near *RING1* (rs213210, rs213209, and rs213208) were found to be associated with GPA in German patients (20). The *HLA-DPB1/RING1* haplotype is strongly associated with GPA in ANCA-positive subjects.

### Non-HLA Region

#### Genes Encoding ANCA Associated Proteins

##### Serpin family A Member 1

*SERPINA1*, located on 14q32.13, encodes α1-antitrypsin, which is the major inhibitor of PR3 and is thought to limit the damage to local tissues (33). In AAV, the genetic variants of *SERPINA1* may lead to the decreased function of α1-antitrypsin, potentially resulting in ANCA generation as PR3 accumulates in tissues, causing the inflammation of blood vessels. The first AAV GWAS reported that the *SERPINA1* Z allele along with the rs7151526 risk allele were significantly associated with AAV and were the most prominent non-*HLA* regions associated with both PR3-ANCA and GPA subgroups (13). Another GWAS confirmed the result with the identification of *SERPINA1* rs28929474, which was associated with AAV (*P* = 3.09 × 10^−12^, OR = 2.18), especially associated with GPA (*P* = 3.53 × 10^−13^, OR = 2.35) (14). Several studies have investigated the role of the Z allele in AAV and showed that heterozygous patients for the Z variant of *SERPINA1* have an increased risk of developing GPA than the general population (34, 35).

##### Proteinase 3 (PRTN3)

*PRTN3*, located on 19p13.3, encodes PR3. PR3 is a neutrophil intracellular protease that is the main antigen of ANCA autoantibodies. PR3 is located on the plasma membrane of a subset of neutrophils and stored in the neutrophil azurophilic granules. The membrane-bound form interacts directly with ANCA (33), decreasing neutrophil activation and endothelial adhesion.

In a Swedish cohort of 79 patients with GPA and 129 controls, the coding and promoter sequences of *PRTN3* were investigated. An association with GPA was demonstrated by *PRTN3* A564G variation in the promoter region affecting a transcription factor-binding site (*P* < 0.00001, OR = 4.2) (36). The association of *PRTN3* has been reported in two GWAS of AAV. Subgroup analysis revealed that *PRTN3* SNP rs62132295 and rs62132293 were significantly associated with AAV, especially PR3-AAV or GPA (*P* = 2.6 × 10^−5^, OR = 0.78; *P* = 8.60 × 10^−11^, OR = 1.29) (13, 14). As observed for the above-mentioned *HLA* association, the strength of the *PRTN3* SNP signal increased in the PR3-ANCA-positive subgroup, independent of the clinical diagnosis (*P* = 2.6 × 10^−7^, OR = 0.73). No association between *PRTN3* and patients with MPA or MPO-AAV was found, which indicates that *PRTN3* only plays a crucial role in the pathogenesis of anti-PR3-positive AAV.

#### Immunoregulatory Genes for AAV

##### Protein Tyrosine Phosphatase, Non-Receptor Type 22

*PTPN22*, located on 1p13.2, encodes lymphoid tyrosine phosphatase (Lyp), a member of the non-receptor class 4 subfamily of the protein tyrosine phosphatase family. *PTPN22* 620W variation causes the substitution of arginine with tryptophan at amino acid residue 620, disturbing the function of *PTPN22*, which is involved in B-cell receptor and T cell receptor signaling, causing autoimmune diseases. It is associated with type 1 diabetes, RA, SLE, and other autoimmune disorders (37, 38).

The 620W allele conferred susceptibility to AAV in a German cohort (*P* = 0.002, OR = 1.75), and the allele frequency was significantly increased in ANCA-positive GPA (*P* < 0.0002) (39). This result has been confirmed in a British cohort (*P* = 1.4 × 10^-4^, OR = 1.40) (40) and an Italian cohort (*P* = 0.005, OR = 1. 91) (41). A meta-analysis of four studies in 1,399 patients of European descent found the association between the *PTPN22* 620W allele and the occurrence and development of AAV (42).

##### Cytotoxic T Lymphocyte-Associated Antigen-4

*CTLA4*, located on 2q33.2, encodes an inhibitory glycoprotein expressed on activated T cells, which transmits an inhibitory signal to T cells. CTLA4 competes with CD28 to bind to CD80 and CD86, which function antagonistically. CTLA4 is a negative regulator of T cell activation and inhibits the immune response, while CD28 transmits a stimulatory signal. The expression of CTLA4 on CD4 T cells is increased in GPA (43). Mutations on this gene have been associated with Graves’ disease, celiac disease, SLE, and other autoimmune diseases.

Although the *CTLA4* SNP did not show genome-wide association in the GWAS of AAV, the association with AAV was found in candidate gene studies in different populations. *CTLA4* rs3087243 showed an association with AAV *in* 641 British AAV cases and 9,115 controls (*P* = 6.4 ×10^-3^, OR=0.84). *CTLA4* exon 1 (+49) and 4 (CT60) polymorphisms showed an association with AAV in another independent British cohorts with 222 cases and 629 controls(*P*_(+49)_ = 0.004, *P*_(CT60)_ = 0.002, respectively) (40, 44). The similar result was confirmed in a Swedish cohort with 101 cases and 200 controls (*P* = 0.049) (45).

##### Toll-Like Receptors

*TLRs*, located on chromosome 3p21.2, encode a family of innate receptors whose specificities are predetermined in the germline. TLRs can recognize microbiological structures and activate immune responses that participate in the development of autoimmune diseases (46). Infection, particularly *Staphylococcus aureus* infection, is considered a potential trigger of AAV, and TLR9 signaling may be involved in disease pathology, favoring models of infectious agents triggering AAV development (47). SNPs in *TLR9* were genotyped in a German cohort comprising 863 patients with AAV and 1,344 healthy controls to investigate the contribution of genetic polymorphisms of *TLR9* to the susceptibility and clinical manifestation. Strong association of *TLR9* genotypes and haplotypes with the subgroups of GPA and MPA was observed, but no association was observed with EGPA. In a cohort of 426 Dutch and British AAV cases, the findings were not replicated (21). TLR9 signaling may be involved in disease pathology, favoring models of infectious agents triggering AAV development.

##### Fc Gamma Receptors

FCGRs are a group of proteins expressed on the surface of different cell types with different affinities for the Fc portions of different IgG subclasses. The key role of Fc receptors is the regulation of inflammatory and immune responses. *FCGR3B* encodes FcgRIIIB, which is expressed by neutrophils and eosinophils and is important in the tethering of neutrophils to immune complexes and the clearance of immune complexes (48). The *FCGR3B* copy number may play a role in the development of SLE or other autoimmune diseases (49).

In a study consisted with two independent cohorts of individuals with GPA from Britain and France, strong association between *FCGR3B* copy numbers and risk of GPA was shown. In British patients, low *FCGR3B* copy number was found to be associated with GPA (*P* = 0.015, OR = 2.46), but in French GPA patients, the association with high *FCGR3B* copy number was identified (*P* = 0.002, OR = 0.28) (50). The study also explored the relationship between *FCGR3B* and MPA in a separate British cohort and observed a significant association between low *FCGR3B* copy number and MPA(*P* = 0.013, OR = 2.56). Later, the results were replicated in a large Chinese cohort with 139 AAV patients and 564 controls (*P* = 0.040, OR = 1.72) with the result that low *FCGR3A* CNVs were significantly associated with AAV susceptibility (*P* = 0.042, OR = 2.64) (51).

Possible associations of SNPs of FCGR2A, FCGR2B, FCGR3A and the FCGR3B haplotype NA1/NA2 and EGPA have been discussed in a recent study, which included 130 EGPA patients and 181 controls. FCGR3B haplotype NA1/NA2 was found to be associated with relapse-free survival of EGPA that relapse-free survival was significantly lower in carriers of FCGR3B haplotype NA1-NA1 than the others (P = 0.029). MPO-ANCA+ EGPA subgroup was also associated with relapse(P = 0.032) while no association was observed in MPO-ANCA- EGPA subgroup(P = 0.68) (52).

##### Interleukin-10

IL-10 is an anti-inflammatory cytokine produced by T helper cells, and its expression increases in patients with AAV (53). Two small studies in German and Caucasian cohorts proposed an association of *IL-10* SNP (–1082) in GPA (54); interestingly, one study also demonstrated a signal for the *IL-10* SNP in 36 patients with MPA (*P* < 1.00× 10^−6^) (55). In a large German cohort of 403 patients with GPA, 103 patients with EGPA and 507 controls, none of the *IL-10* polymorphisms were associated with GPA, but *IL-10* -3575/-1082/-592 TAC haplotype was highly significantly associated with ANCA-negative EGPA (*P* = 3.00 × 10^−5^, OR = 2.16) (56).

##### Interleukin 2 Receptor Subunit Alpha

*IL2RA* encodes the high-affinity IL-2 receptor, which is represented not only on the surface of T cells, but also on activated B cells, NK cells, and monocytes (57). The IL2-IL2RA pathway is critical for the physiological function of the immune system; survival, proliferation, and activation of T cells and development of a normal Treg repertoire (58). An association has been reported in a UK cohort of 670 patients with AAV for the *IL2RA* SNP rs41295061 (*P* = 1.22× 10^−2^) (59), but no correlation between *IL12RA* and AAV was found during the replication stage of the first GWAS validation.

#### Other Susceptible Genes

##### Defensin Beta 4A

DEFB4 is an antibiotic peptide. The copy number variation of *DEFB4* reportedly causes susceptibility to inflammatory disorders (60). Higher *DEFB4* copy number was demonstrated to be significantly higher in a small cohort of 112 Chinese patients with AAV than in controls, which supported the role of the defensin system in autoimmunity (61).

##### Rho GTPase-Activating Protein 18

ARHGAP18 belongs to a family of Rho GTPase-activating proteins that modulate cell signaling. The subgroup analysis of the first GWAS for AAV revealed that *ARHGAP18* was associated with GPA (*P* = 3.3 × 10^-7^, OR = 0.84) and PR3-ANCA AAV (*P* = 5.2 ×10^-8^, OR = 0.87) (13).

##### BCL2-Like11 and MIR4435-2HG(MORRBID)

*BCL2L11* encodes BIM, which belongs to the BCL-2 protein family and is crucial for controlling apoptosis, immune homeostasis, and autoimmune diseases (62, 63). *MORRBID* encodes a long non-coding RNA that regulates BIM transcription, controls eosinophil apoptosis, and is dysregulated in hypereosinophilic syndrome (64). In the GWAS of EGPA, SNPs near *BCL2L11* and *MORRBID* were identified to be associated with EGPA (*P* = 9 × 10^-11^, OR = 1.66) (16).

##### Thymic Stromal Lymphopoietin

*TSLP* is located on 5q22.1. Its product, TSLP, is released by stromal and epithelial cells in response to inflammatory stimuli, which drives eosinophilia and enhances TH2 responses that are associated with immunity in various inflammatory diseases, including asthma, allergic inflammation, and chronic obstructive pulmonary disease (65). *TSLP* SNP rs1837253 which lies immediately upstream of TSLP was observed to be EGPA susceptibility variant (*P* = 5.2 × 10^-11^, OR = 1.42) (16).

##### ETS Proto-Oncogene 1

*ETS1*, located on chromosome 11q24.3, encodes a member of the ETS family of transcription factors. ETS1 inhibits the differentiation of Th17 cells and induces the development of Tregs. Genetic variation may cause an increase in Th17 in patients with GPA, causing inflammation of small vessels. A GWAS in a Chinese population reported that *ETS1* SNP rs1128334 was significantly associated with SLE (66). In order to observe whether this gene was associated with other autoimmune diseases, a Japanese study which contained 466 patients with AAV and 1099 healthy controls was carried out. *ETS1* SNP rs1128334 was genotyped and found to be associated with GPA (*P* = 0.030, OR = 1.54) and PR3-ANCA AAV (*P* = 0.021, OR = 1.72) (67).

##### Telomerase Reverse Transcriptase and Desmoplakin

TERT is the catalytic subunit of telomerase and contributes to the maintenance of telomere length (68). *TERT* rs2736100A is associated with shorter leukocyte telomere length (69). TERT also has an anti-apoptotic effect, and a decreased expression of TERT associated with the risk allele may result in the enhancement of apoptosis (70). With the evidence that a proportion of T cells show short telomeres in patients with GPA and an enhancement in neutrophil apoptosis in patients with AAV, *TERT* has been proposed as a susceptibility gene for AAV (71). In a Japanese cohort comprising 544 patients with AAV and 5558 controls, the frequency of *TERT* rs2736100A alleles was significantly increased in MPA (*P* = 2.3 × 10^−2^, OR= 1.38) and MPO-AAV (*P* = 0.15× 10^−2^, OR = 1.33) (72). *DSP* rs2076295G showed similar results to *TERT* rs2736100A (*P* = 0.69× 10^-2^, OR = 1.32) in MPO-AAV (*P* = 0.011, OR = 1.26) (72). DSP reportedly modulates Wnt/β-catenin signaling, which is involved in cell proliferation, differentiation, immune response, and carcinogenesis. Wnt signaling plays a role in autoimmune diseases and may also be associated with the onset of AAV.

All the genes associated with AAV were summarized in **Table 3**.

**Table 3:** Summary of genetic associations with antineutrophil cytoplasmic antibody (ANCA)-associated vasculitis excluding those derived from genome-wide association studies.

Gene	Variation	Disease	Populations
*HLA*	DQA1*0302, DQB1*0303, DPB1*04:01, DRB1*0901, DRB1*1101, DRB1*13:02, HLA–DPA1, HLA–DQA2	AAV (GPA, MPA, EGPA)	Worldwide
*RING*	rs213210, rs213209, and rs213208	GPA	German
*RXRB*	rs6531	GPA	German
*CD226*	rs763361	GPA	German
*CTLA4*	rs3087243, CT60	GPA, MPA and EGPA	British, Swedish
*DEFB4*	High CNVs	AAV	Chinese
*DSP*	rs2076295G	MPA	Japanese
*ETS1*	rs1128334	GPA	Japanese
*FCGR3B*	Low CNVs, High CNVs	AAV	French, British, Chinese
*IL10*	IL10 -3575/-1082/-592 TAC haplotype	EGPA( ANCA- )	German
*IL2RA*	rs41295061	AAV	British
*MUC5B*	rs35705950	AAV-ILD	Japanese
*PTPN22*	rs2476601, C1858T	GPA, MPA	German, Italian, British
*TLR-9*	rs352162, rs352140, rs352139 CTC, TCT	GPA, MPA, EGPA	German, British, Dutch
*TERT*	rs2736100A	MPA	Japanese

## Conclusion

In the past 20 years, genetic studies, including GWAS and candidate gene studies, have identified numerous key loci that are associated with the risk of AAV. These genes have not only provided novel etiological clues to deepen our understanding of AAV but also novel therapeutic and prophylactic biomarkers for new approaches to treat and prevent AVV. Biomarker-targeting therapies and preventing recurrence in the future may be more effective. With the increasing number of GWAS being conducted, it is desirable to combine these findings and improve the statistical power. To date, GWAS for AAV have been performed in the European population; in the future, more GWAS with large samples in different populations and different subgroups of AAV are required to reveal the pathogenesis of the disease and identify reliable biomarkers for precision medicine.

## Author Contributions

YS is the investigator for the review article and has contributed to the concept and planning of the article, collection of data, and reporting of the work described. WL, HH, MC, and TY contributed to the collection of data, and reporting of the work described. All authors contributed to the article and approved the submitted version.

## Conflict of Interest

The authors declare that the research was conducted in the absence of any commercial or financial relationships that could be construed as a potential conflict of interest.

## References

[1] Hunter RW Welsh N Farrah TE Gallacher PJ Dhaun N. ANCA associated vasculitis. BMJ (2020) 369:m1070. 10.1136/bmj.m1070 32291255 PMC7179255

[2] Kitching AR Anders HJ Basu N Brouwer E Gordon J Jayne DR. ANCA-associated vasculitis. Nat Rev Dis Primers (2020) 6:71. 10.1038/s41572-020-0204-y 32855422

[3] Berden A Goceroglu A Jayne D Luqmani R Rasmussen N Bruijn JA. Diagnosis and management of ANCA associated vasculitis. BMJ (2012) 344:e26. 10.1136/bmj.e26 22250224

[4] Ugarte-Gil MF Espinoza LR. Genetics of ANCA-associated Vasculitides. Curr Rheumatol Rep (2014) 16:428. 10.1007/s11926-014-0428-5 24828480

[5] Berti A Cornec D Crowson CS Specks U Matteson EL. The Epidemiology of Antineutrophil Cytoplasmic Autoantibody-Associated Vasculitis in Olmsted County, Minnesota: A Twenty-Year US Population-Based Study. Arthritis Rheumatol (2017) 69:2338–50. 10.1002/art.40313 PMC5711593 28881446

[6] Chen M Kallenberg CG. The environment, geoepidemiology and ANCA-associated vasculitides. Autoimmun Rev (2010) 9:A293–8. 10.1016/j.autrev.2009.10.008 19892038

[7] Prendecki M Cairns T Pusey CD. Familial vasculitides: granulomatosis with polyangitis and microscopic polyangitis in two brothers with differing anti-neutrophil cytoplasm antibody specificity. Clin Kidney J (2016) 9:429–31. 10.1093/ckj/sfw016 PMC4886912 27274829

[8] Papiha SS Murty GE Ad’Hia A Mains BT Venning M. Association of Wegener’s granulomatosis with HLA antigens and other genetic markers. Ann Rheum Dis (1992) 51:246–8. 10.1136/ard.51.2.246 PMC1005667 1550412

[9] Bonatti F Reina M Neri TM Martorana D. Genetic Susceptibility to ANCA-Associated Vasculitis: State of the Art. Front Immunol (2014) 5:577. 10.3389/fimmu.2014.00577 25452756 PMC4233908

[10] Wallace ZS Stone JH. Personalized Medicine in ANCA-Associated Vasculitis ANCA Specificity as the Guide? Front Immunol (2019) 10:2855. 10.3389/fimmu.2019.02855 31867013 PMC6909331

[11] Salama AD. Genetics and pathogenesis of small-vessel vasculitis. Best Pract Res Clin Rheumatol (2018) 32:21–30. 10.1016/j.berh.2018.10.002 30526895

[12] Alberici F Martorana D Vaglio A. Genetic aspects of anti-neutrophil cytoplasmic antibody-associated vasculitis. Nephrol Dial Transplant (2015) 30 Suppl 1:i37–45. 10.1093/ndt/gfu386 25523449

[13] Lyons PA Rayner TF Trivedi S Holle JU Watts RA Jayne DR. Genetically distinct subsets within ANCA-associated vasculitis. N Engl J Med (2012) 367:214–23. 10.1056/NEJMoa1108735 PMC3773907 22808956

[14] Merkel PA Xie G Monach PA Ji X Ciavatta DJ Byun J. Identification of Functional and Expression Polymorphisms Associated With Risk for Antineutrophil Cytoplasmic Autoantibody-Associated Vasculitis. Arthritis Rheumatol (2017) 69:1054–66. 10.1002/art.40034 PMC5434905 28029757

[15] Xie G Roshandel D Sherva R Monach PA Lu EY Kung T. Association of granulomatosis with polyangiitis (Wegener’s) with HLA-DPB1*04 and SEMA6A gene variants: evidence from genome-wide analysis. Arthritis Rheum (2013) 65:2457–68. 10.1002/art.38036 PMC4471994 23740775

[16] Lyons PA Peters JE Alberici F Liley J Coulson RMR Astle W. Genome-wide association study of eosinophilic granulomatosis with polyangiitis reveals genomic loci stratified by ANCA status. Nat Commun (2019) 10:5120. 10.1038/s41467-019-12515-9 31719529 PMC6851141

[17] Rahmattulla C Mooyaart AL van Hooven D Schoones JW Bruijn JA Dekkers OM. Genetic variants in ANCA-associated vasculitis: a meta-analysis. Ann Rheum Dis (2016) 75:1687–92. 10.1136/annrheumdis-2015-207601 26443607

[18] Jagiello P Gencik M Arning L Wieczorek S Kunstmann E Csernok E. New genomic region for Wegener’s granulomatosis as revealed by an extended association screen with 202 apoptosis-related genes. Hum Genet (2004) 114:468–77. 10.1007/s00439-004-1092-z 14968360

[19] Szyld P Jagiello P Csernok E Gross WL Epplen JT. On the Wegener granulomatosis associated region on chromosome 6p21.3. BMC Med Genet (2006) 7:21. 10.1186/1471-2350-7-21 16526951 PMC1431512

[20] Heckmann M Holle JU Arning L Knaup S Hellmich B Nothnagel M. The Wegener’s granulomatosis quantitative trait locus on chromosome 6p21.3 as characterised by tagSNP genotyping. Ann Rheum Dis (2008) 67:972–9. 10.1136/ard.2007.077693 17967832

[21] Husmann CA Holle JU Moosig F Mueller S Wilde B Cohen Tervaert JW. Genetics of toll like receptor 9 in ANCA associated vasculitides. Ann Rheum Dis (2014) 73:890–6. 10.1136/annrheumdis-2012-202803 23592712

[22] Ghodke Y Joshi K Chopra A Patwardhan B. HLA and disease. Eur J Epidemiol (2005) 20:475–88. 10.1007/s10654-005-5081-x 16121756

[23] Matzaraki V Kumar V Wijmenga C Zhernakova A. The MHC locus and genetic susceptibility to autoimmune and infectious diseases. Genome Biol (2017) 18:76. 10.1186/s13059-017-1207-1 28449694 PMC5406920

[24] Wu Z Wu Q Xu J Chen S Sun F Li P. HLA-DPB1 variant rs3117242 is associated with anti-neutrophil cytoplasmic antibody-associated vasculitides in a Han Chinese population. Int J Rheum Dis (2017) 20:1009–15. 10.1111/1756-185X.12561 26014903

[25] Tsuchiya N Kobayashi S Kawasaki A Kyogoku C Arimura Y Yoshida M. Genetic background of Japanese patients with antineutrophil cytoplasmic antibody-associated vasculitis: association of HLA-DRB1*0901 with microscopic polyangiitis. J Rheumatol (2003) 30:1534–40. 12858454

[26] Kawasaki A Hasebe N Hidaka M Hirano F Sada KE Kobayashi S. Protective Role of HLA-DRB1*13:02 against Microscopic Polyangiitis and MPO-ANCA-Positive Vasculitides in a Japanese Population: A Case-Control Study. PloS One (2016) 11:e0154393. 10.1371/journal.pone.0154393 27166610 PMC4868057

[27] Tsuchiya N Kobayashi S Hashimoto H Ozaki S Tokunaga K. Association of HLA-DRB1*0901-DQB1*0303 haplotype with microscopic polyangiitis in Japanese. Genes Immun (2006) 7:81–4. 10.1038/sj.gene.6364262 16208405

[28] Wang HY Cui Z Pei ZY Fang SB Chen SF Zhu L. Risk HLA class II alleles and amino acid residues in myeloperoxidase-ANCA-associated vasculitis. Kidney Int (2019) 96:1010–9. 10.1016/j.kint.2019.06.015 31471160

[29] Wieczorek S Hellmich B Gross WL Epplen JT. Associations of Churg-Strauss syndrome with the HLA-DRB1 locus, and relationship to the genetics of antineutrophil cytoplasmic antibody-associated vasculitides: comment on the article by Vaglio et al. Arthritis Rheum (2008) 58:329–30. 10.1002/art.23209 18163478

[30] Vaglio A Martorana D Maggiore U Grasselli C Zanetti A Pesci A. HLA-DRB4 as a genetic risk factor for Churg-Strauss syndrome. Arthritis Rheum (2007) 56:3159–66. 10.1002/art.22834 17763415

[31] Chang DY Luo H Zhou XJ Chen M Zhao MH. Association of HLA genes with clinical outcomes of ANCA-associated vasculitis. Clin J Am Soc Nephrol (2012) 7:1293–9. 10.2215/CJN.13071211 PMC3408128 22595829

[32] Hilhorst M Arndt F Joseph Kemna M Wieczorek S Donner Y Wilde B. HLA-DPB1 as a Risk Factor for Relapse in Antineutrophil Cytoplasmic Antibody-Associated Vasculitis: A Cohort Study. Arthritis Rheumatol (2016) 68:1721–30. 10.1002/art.39620 26866715

[33] Campbell EJ Campbell MA Owen CA. Bioactive proteinase 3 on the cell surface of human neutrophils: quantification, catalytic activity, and susceptibility to inhibition. J Immunol (2000) 165:3366–74. 10.4049/jimmunol.165.6.3366 10975855

[34] Elzouki AN Segelmark M Wieslander J Eriksson S. Strong link between the alpha 1-antitrypsin PiZ allele and Wegener’s granulomatosis. J Intern Med (1994) 236:543–8. 10.1111/j.1365-2796.1994.tb00842.x 7964431

[35] Baslund B Szpirt W Eriksson S Elzouki AN Wiik A Wieslander J. Complexes between proteinase 3, alpha 1-antitrypsin and proteinase 3 anti-neutrophil cytoplasm autoantibodies: a comparison between alpha 1-antitrypsin PiZ allele carriers and non-carriers with Wegener’s granulomatosis. Eur J Clin Invest (1996) 26:786–92. 10.1046/j.1365-2362.1996.2070553.x 8889441

[36] Gencik M Meller S Borgmann S Fricke H. Proteinase 3 gene polymorphisms and Wegener’s granulomatosis. Kidney Int (2000) 58:2473–7. 10.1046/j.1523-1755.2000.00430.x 11115080

[37] Lamsyah H Rueda B Baassi L Elaouad R Bottini N Sadki K. Association of PTPN22 gene functional variants with development of pulmonary tuberculosis in Moroccan population. Tissue Antigens (2009) 74:228–32. 10.1111/j.1399-0039.2009.01304.x 19563523

[38] Vang T Congia M Macis MD Musumeci L Orru V Zavattari P. Autoimmune-associated lymphoid tyrosine phosphatase is a gain-of-function variant. Nat Genet (2005) 37:1317–9. 10.1038/ng1673 16273109

[39] Jagiello P Aries P Arning L Wagenleiter SE Csernok E Hellmich B. The PTPN22 620W allele is a risk factor for Wegener’s granulomatosis. Arthritis Rheum (2005) 52:4039–43. 10.1002/art.21487 16320352

[40] Carr EJ Niederer HA Williams J Harper L Watts RA Lyons PA. Confirmation of the genetic association of CTLA4 and PTPN22 with ANCA-associated vasculitis. BMC Med Genet (2009) 10:121. 10.1186/1471-2350-10-121 19951419 PMC3224698

[41] Martorana D Maritati F Malerba G Bonatti F Alberici F Oliva E. PTPN22 R620W polymorphism in the ANCA-associated vasculitides. Rheumatology (Oxford) (2012) 51:805–12. 10.1093/rheumatology/ker446 22237046

[42] Cao Y Liu K Tian Z Hogan SL Yang J Poulton CJ. PTPN22 R620W polymorphism and ANCA disease risk in white populations: a metaanalysis. J Rheumatol (2015) 42:292–9. 10.3899/jrheum.131430 PMC4314360 25448792

[43] Steiner K Moosig F Csernok E Selleng K Gross WL Fleischer B. Increased expression of CTLA-4 (CD152) by T and B lymphocytes in Wegener’s granulomatosis. Clin Exp Immunol (2001) 126:143–50. 10.1046/j.1365-2249.2001.01575.x PMC1906160 11678911

[44] Kamesh L Heward JM Williams JM Gough SC Chavele KM Salama A. CT60 and +49 polymorphisms of CTLA 4 are associated with ANCA-positive small vessel vasculitis. Rheumatology (Oxford) (2009) 48:1502–5. 10.1093/rheumatology/kep280 19815671

[45] Persson U Gullstrand B Pettersson A Sturfelt G Truedsson L Segelmark M. A candidate gene approach to ANCA-associated vasculitis reveals links to the C3 and CTLA-4 genes but not to the IL1-Ra and Fcgamma-RIIa genes. Kidney Blood Press Res (2013) 37:641–8. 10.1159/000355744 24356554

[46] Marshak-Rothstein A. Toll-like receptors in systemic autoimmune disease. Nat Rev Immunol (2006) 6:823–35. 10.1038/nri1957 PMC7097510 17063184

[47] Popa ER Stegeman CA Abdulahad WH van der Meer B Arends J Manson WM. Staphylococcal toxic-shock-syndrome-toxin-1 as a risk factor for disease relapse in Wegener’s granulomatosis. Rheumatology (Oxford) (2007) 46:1029–33. 10.1093/rheumatology/kem022 17409134

[48] Smith KG Clatworthy MR. FcgammaRIIB in autoimmunity and infection: evolutionary and therapeutic implications. Nat Rev Immunol (2010) 10:328–43. 10.1038/nri2762 PMC4148599 20414206

[49] Mueller M Barros P Witherden AS Roberts AL Zhang Z Schaschl H. Genomic pathology of SLE-associated copy-number variation at the FCGR2C/FCGR3B/FCGR2B locus. Am J Hum Genet (2013) 92:28–40. 10.1016/j.ajhg.2012.11.013 23261299 PMC3542466

[50] Fanciulli M Norsworthy PJ Petretto E Dong R Harper L Kamesh L. FCGR3B copy number variation is associated with susceptibility to systemic, but not organ-specific, autoimmunity. Nat Genet (2007) 39:721–3. 10.1038/ng2046 PMC2742197 17529978

[51] Qi Y Zhou X Bu D Hou P Lv J Zhang H. Low copy numbers of FCGR3A and FCGR3B associated with Chinese patients with SLE and AASV. Lupus (2017) 26:1383–9. 10.1177/0961203317700485 28355982

[52] Alberici F Bonatti F Adorni A Daminelli G Sinico RA Gregorini G. FCGR3B polymorphism predicts relapse risk in eosinophilic granulomatosis with polyangiitis. Rheumatology (Oxford) (2020) 59:3563–6. 10.1093/rheumatology/keaa134 32375167

[53] Wang Y Zhang S Zhang N Feng M Liang Z Zhao X. Reduced activated regulatory T cells and imbalance of Th17/activated Treg cells marks renal involvement in ANCA-associated vasculitis. Mol Immunol (2020) 118:19–29. 10.1016/j.molimm.2019.11.010 31837507

[54] Murakozy G Gaede KI Ruprecht B Gutzeit O Schurmann M Schnabel A. Gene polymorphisms of immunoregulatory cytokines and angiotensin-converting enzyme in Wegener’s granulomatosis. J Mol Med (Berl) (2001) 79:665–70. 10.1007/s001090100263 11715070

[55] Bartfai Z Gaede KI Russell KA Murakozy G Muller-Quernheim J Specks U. Different gender-associated genotype risks of Wegener’s granulomatosis and microscopic polyangiitis. Clin Immunol (2003) 109:330–7. 10.1016/s1521-6616(03)00211-0 14697748

[56] Wieczorek S Hellmich B Arning L Moosig F Lamprecht P Gross WL. Functionally relevant variations of the interleukin-10 gene associated with antineutrophil cytoplasmic antibody-negative Churg-Strauss syndrome, but not with Wegener’s granulomatosis. Arthritis Rheum (2008) 58:1839–48. 10.1002/art.23496 18512809

[57] Caruso C Candore G Cigna D Colucci AT Modica MA. Biological significance of soluble IL-2 receptor. Mediators Inflamm (1993) 2:3–21. 10.1155/S0962935193000018 18475497 PMC2365387

[58] Fontenot JD Rasmussen JP Gavin MA Rudensky AY. A function for interleukin 2 in Foxp3-expressing regulatory T cells. Nat Immunol (2005) 6:1142–51. 10.1038/ni1263 16227984

[59] Carr EJ Clatworthy MR Lowe CE Todd JA Wong A Vyse TJ. Contrasting genetic association of IL2RA with SLE and ANCA-associated vasculitis. BMC Med Genet (2009) 10:22. 10.1186/1471-2350-10-22 19265545 PMC2662820

[60] Hollox EJ Huffmeier U Zeeuwen PL Palla R Lascorz J Rodijk-Olthuis D. Psoriasis is associated with increased beta-defensin genomic copy number. Nat Genet (2008) 40:23–5. 10.1038/ng.2007.48 PMC2447885 18059266

[61] Zhou XJ Cheng FJ Lv JC Luo H Yu F Chen M. Higher DEFB4 genomic copy number in SLE and ANCA-associated small vasculitis. Rheumatology (Oxford) (2012) 51:992–5. 10.1093/rheumatology/ker419 22302058

[62] Bouillet P Purton JF Godfrey DI Zhang LC Coultas L Puthalakath H. BH3-only Bcl-2 family member Bim is required for apoptosis of autoreactive thymocytes. Nature (2002) 415:922–6. 10.1038/415922a 11859372

[63] Enders A Bouillet P Puthalakath H Xu Y Tarlinton DM Strasser A. Loss of the pro-apoptotic BH3-only Bcl-2 family member Bim inhibits BCR stimulation-induced apoptosis and deletion of autoreactive B cells. J Exp Med (2003) 198:1119–26. 10.1084/jem.20030411 PMC2194219 14517273

[64] Kotzin JJ Spencer SP McCright SJ Kumar DBU Collet MA Mowel WK. The long non-coding RNA Morrbid regulates Bim and short-lived myeloid cell lifespan. Nature (2016) 537:239–43. 10.1038/nature19346 PMC5161578 27525555

[65] Ziegler SF Roan F Bell BD Stoklasek TA Kitajima M Han H. The biology of thymic stromal lymphopoietin (TSLP). Adv Pharmacol (2013) 66:129–55. 10.1016/B978-0-12-404717-4.00004-4 PMC4169878 23433457

[66] Yang W Shen N Ye DQ Liu Q Zhang Y Qian XX. Genome-wide association study in Asian populations identifies variants in ETS1 and WDFY4 associated with systemic lupus erythematosus. PloS Genet (2010) 6:e1000841. 10.1371/journal.pgen.1000841 20169177 PMC2820522

[67] Kawasaki A Yamashita K Hirano F Sada KE Tsukui D Kondo Y. Association of ETS1 polymorphism with granulomatosis with polyangiitis and proteinase 3-anti-neutrophil cytoplasmic antibody positive vasculitis in a Japanese population. J Hum Genet (2018) 63:55–62. 10.1038/s10038-017-0362-2 29167552

[68] Nagpal N Agarwal S. Telomerase RNA processing: Implications for human health and disease. Stem Cells (2020). 10.1002/stem.3270 PMC7917152 32875693

[69] Codd V Nelson CP Albrecht E Mangino M Deelen J Buxton JL. Identification of seven loci affecting mean telomere length and their association with disease. Nat Genet (2013) 45:422–7, 427e1-2. 10.1038/ng.2528 23535734 PMC4006270

[70] Sung YH Choi YS Cheong C Lee HW. The pleiotropy of telomerase against cell death. Mol Cells (2005) 19:303–9. 15995345

[71] Vogt S Iking-Konert C Hug F Andrassy K Hansch GM. Shortening of telomeres: Evidence for replicative senescence of T cells derived from patients with Wegener’s granulomatosis. Kidney Int (2003) 63:2144–51. 10.1046/j.1523-1755.2003.00037.x 12753301

[72] Kawasaki A Namba N Sada KE Hirano F Kobayashi S Nagasaka K. Association of TERT and DSP variants with microscopic polyangiitis and myeloperoxidase-ANCA positive vasculitis in a Japanese population: a genetic association study. Arthritis Res Ther (2020) 22:246. 10.1186/s13075-020-02347-0 33076992 PMC7574242

